# Vessel diameter as a potential predictor of pulsed dye laser response in port wine stain: a retrospective exploratory reflectance confocal microscopy study

**DOI:** 10.1007/s10103-026-04906-4

**Published:** 2026-06-10

**Authors:** Xuehui Ran, Ying Shang, Xia Yang, Yufeng Wang, Hao Peng, Lingyue Shen

**Affiliations:** 1 Department of Oral & Maxillofacial-Head & Neck Oncology, Shanghai Jiao Tong University School of Medicine; College of Stomatology, Shanghai Jiao Tong University, National Center for Stomatology; National Clinical Research Center for Oral Diseases; Shanghai Key Laboratory of Stomatology; Shanghai Research Institute of Stomatology., Shanghai Ninth People’s Hospital, Shanghai, China; 2 Department of Laser and Aesthetic Medicine, Shanghai Ninth People’s Hospital, Shanghai, China; 3 Department of Oral Medicine, Shanghai Ninth People’s Hospital, Shanghai Jiao Tong University School of Medicine; College of Stomatology, Shanghai Jiao Tong University; National Center for Stomatology; National Clinical Research Center for Oral Diseases; Shanghai Key Laboratory of Stomatology; Shanghai Research Institute of Stomatology., Shanghai Ninth People’s Hospital, Shanghai, China

**Keywords:** Reflectance confocal microscopy (RCM), Port wine stain (PWS), Pulsed dye laser (PDL), Vessel diameter, Treatment resistance

## Abstract

To investigate whether noninvasive reflectance confocal microscopy (RCM)–derived vascular parameters can serve as objective predictors of therapeutic response to pulsed dye laser (PDL) treatment in patients with port wine stain (PWS), given the wide variability in clinical outcomes. We retrospectively reviewed the records of 240 patients with facial PWS who underwent RCM imaging before a scheduled PDL session, yielding their image datasets stratified by treatment history. The superficial vessel depth (SVD), maximum vessel diameter (MVD), and vessel density (VD) were quantified. Among 25 treatment-naïve patients with follow-up evaluations, treatment efficacy after a single PDL session was graded on a standardized quartile scale. Associations between RCM parameters and clinical outcomes were evaluated using nonparametric tests and Fisher’s exact test, as appropriate. A longitudinal analysis was performed in 14 patients with available pre- and post-treatment RCM data. Vascular parameters differed significantly among groups stratified by prior PDL sessions. With increasing treatment sessions, SVD increased whereas MVD decreased significantly (both p < 0.05), while VD showed no significant difference. Among treatment-naïve patients, baseline MVD > 40 μm was associated with a significantly higher response rate than MVD ≤ 40 μm (p < 0.001). In the longitudinal subset, effective responders showed a significant post-treatment reduction in MVD, whereas ineffective responders did not. Baseline MVD assessed by RCM may serve as a potential imaging biomarker for predicting PDL response in patients with PWS. Larger vessels were associated with more favorable clinical improvement, while smaller vessels tended to show poorer response. These preliminary findings require validation in larger prospective cohorts before clinical implementation.

## Introduction

Port wine stain (PWS) is a common congenital capillary malformation with an incidence of 0.3–0.5% in newborns [1]. PWS typically appears at birth as pink or red macules, which may darken, thicken, and develop nodules over time [2]. Approximately 80% of PWS lesions occur on the face and neck, leading to low self-esteem and disease-related stigma, which may significantly affect patients’ psychological health and quality of life [3, 4]. Current therapies mainly include pulsed dye laser (PDL) treatment (the gold standard) and photodynamic therapy [5–7]. Although some patients can achieve great blanching of PWS lesions after PDL treatment, many present limited improvements, and some even fail to respond at all [8]. It has been reported that only 21% of patients achieve a good response (clearance rate > 75%), and the efficacy of PDL treatment has not improved over the last 30 years [9]. Multiple PDL treatments are usually needed, but treatment outcomes are difficult to predict because of interindividual variability, which can cause considerable anxiety for both patients and clinicians. The possible mechanisms underlying the poor response to laser therapy in patients with PWS have been analyzed, such as the age of the patients, anatomical location, skin thickness, characteristics of PWS vessels (depth, diameter, and wall thickness), and number of treatments [10]. Initial histological studies were conducted to explore the mechanisms underlying variable PDL treatment responses. Fiskerstrand et al. reported that poor responders had significantly smaller and deeper vessels [11]. Therefore, PWS blood vessel characteristics may be crucial factors for inadequate treatment outcomes. Owing to the invasiveness and limited practicality of histopathological examination, objective evaluation of PWS in routine settings remains challenging, underscoring the need for reliable, noninvasive imaging modalities.

Recently, several noninvasive methods for predicting the efficacy of PWS treatment have been reported, such as dermoscopy, high-frequency ultrasound, optical coherence tomography, laser Doppler imaging, and reflectance confocal microscopy (RCM) [12]. Among these, RCM has gained particular attention because of its high resolution, which is comparable to histopathology, and its ability to provide real-time [13], noninvasive imaging of skin lesions at cellular-level resolution [14], allowing visualization and quantitative analysis of blood vessels [15]. Physicians have already used RCM to detect vascular lesions in patients with PWS and to investigate the correlation between vascular characteristics and PDL therapy outcomes. Earlier studies [16] have suggested that vessels with higher flow, larger diameters, and greater depth tend to show poorer clearance following PDL therapy; however, there is still a limited body of research quantitatively correlating in vivo RCM vascular parameters with clinical outcomes of PDL treatment in PWS patients.

In this study, a larger sample size was used to quantify vascular characteristics in PWS lesions via RCM, and their correlation with PDL treatment efficacy was analyzed. This study aims to provide clinicians with objective reference indicators to optimize treatment protocols and improve the predictive accuracy of efficacy models.

## Materials and methods

### Patients and study design

This retrospective study reviewed the medical records of patients with facial PWS who presented to the Department of Laser and Aesthetic Medicine and the Department of Head, Neck and Maxillofacial Oncology at an academic tertiary referral center between December 2022 and March 2025. The diagnosis was confirmed by a senior oral and maxillofacial surgeon in accordance with the Guidelines for the Diagnosis and Treatment of Hemangioma and Vascular Malformation (2019 edition) [17].

**Fig. 1:**
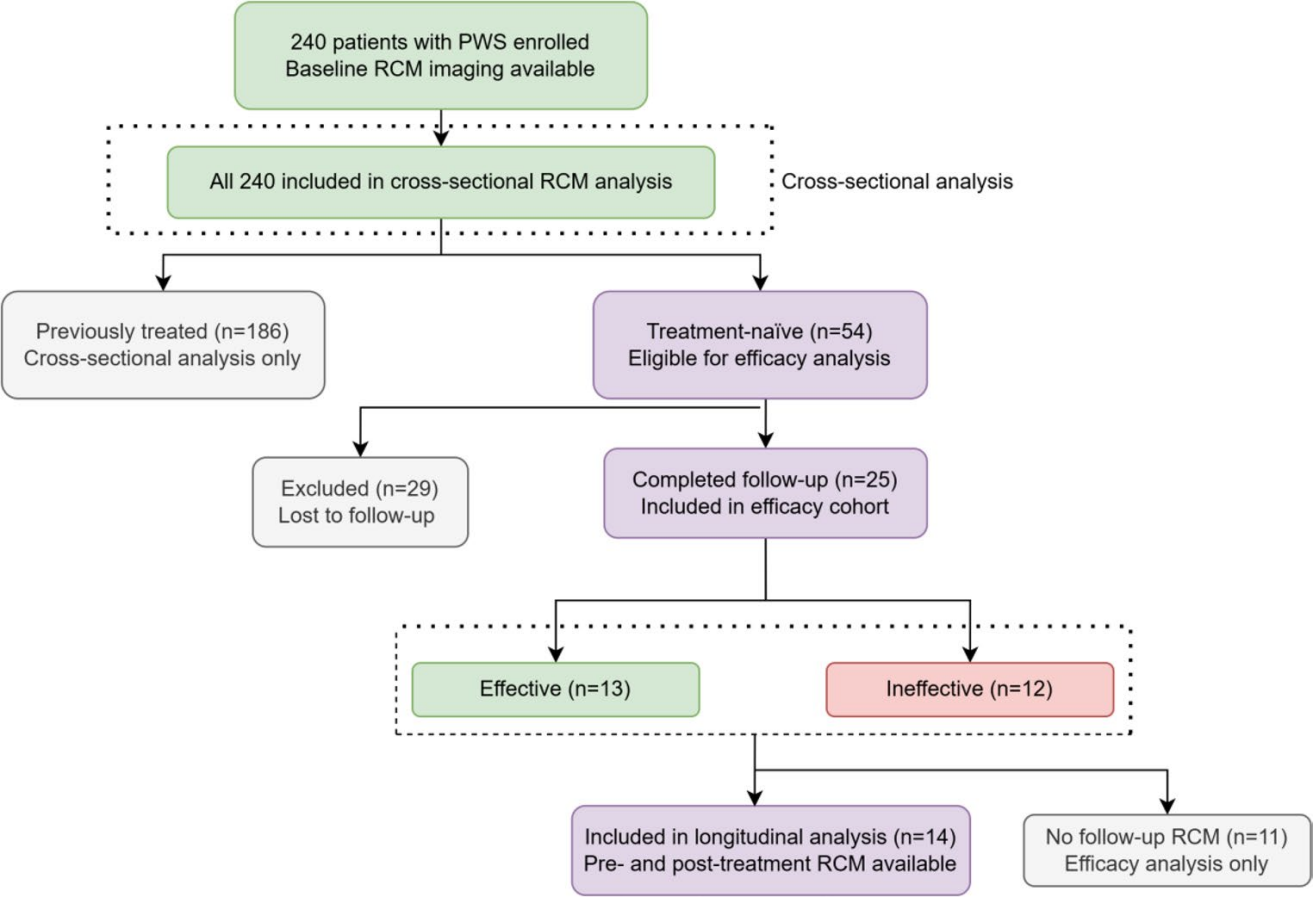
Patient flow diagram. A total of 240 patients with PWS were enrolled and included in the cross-sectional RCM analysis. Of these, 186 previously treated patients were included in the cross-sectional analysis only. The remaining 54 treatment-naïve patients were eligible for the efficacy analysis; 29 were excluded, leaving 25 in the efficacy cohort. These 25 patients were dichotomized into effective (*n* = 13) and ineffective (*n* = 12) groups. Of the 25, 14 had pre- and post-treatment RCM data available and were included in the longitudinal analysis; the remaining 11 were included in the efficacy analysis only. Abbreviations: PWS, port wine stain

Patients who met the predefined inclusion and exclusion criteria were included in the cross-sectional RCM analysis. Treatment-naïve patients with available standardized clinical photographs at baseline and follow-up were further included in the efficacy analysis. A subset of these patients with paired pre- and post-treatment RCM images obtained before and approximately 2 months after the first PDL session was included in the longitudinal analysis. The patient selection process is shown in Fig. 1.

### RCM imaging and treatment procedure

All PWS lesions were treated with a 595-nm pulsed dye laser (Candela, Inc., Westford, MA). Energy fluences of 6.0–8.25 J/cm², pulse durations of 0.45–1.5 ms, and a 10-mm spot size were used. Treatment parameters were individualized by the treating physician based on lesion characteristics (color, location, Fitzpatrick skin type) and patient tolerance, consistent with standard clinical practice. The entire lesion was treated with no overlap between adjacent spots. Patients received PDL treatments at intervals of approximately 2 months, and an RCM examination was performed immediately before each PDL session.

Baseline RCM imaging was performed for every patient using the VivaScope 3000 system (Caliber I.D., Rochester, NY, USA) at three predefined sites within the lesion immediately before the PDL session, regardless of prior treatment history. The three imaging sites were standardized as the center of the lesion and two peripheral sites (superior and inferior borders), selected to capture representative distribution of the lesion vasculature. For patients who returned for follow-up, the same anatomical landmarks were used for repeat imaging to ensure consistency. All image sets were analyzed retrospectively by one trained investigator (X.R.), who was blinded to clinical outcome data at the time of RCM assessment.

**Fig. 2:**
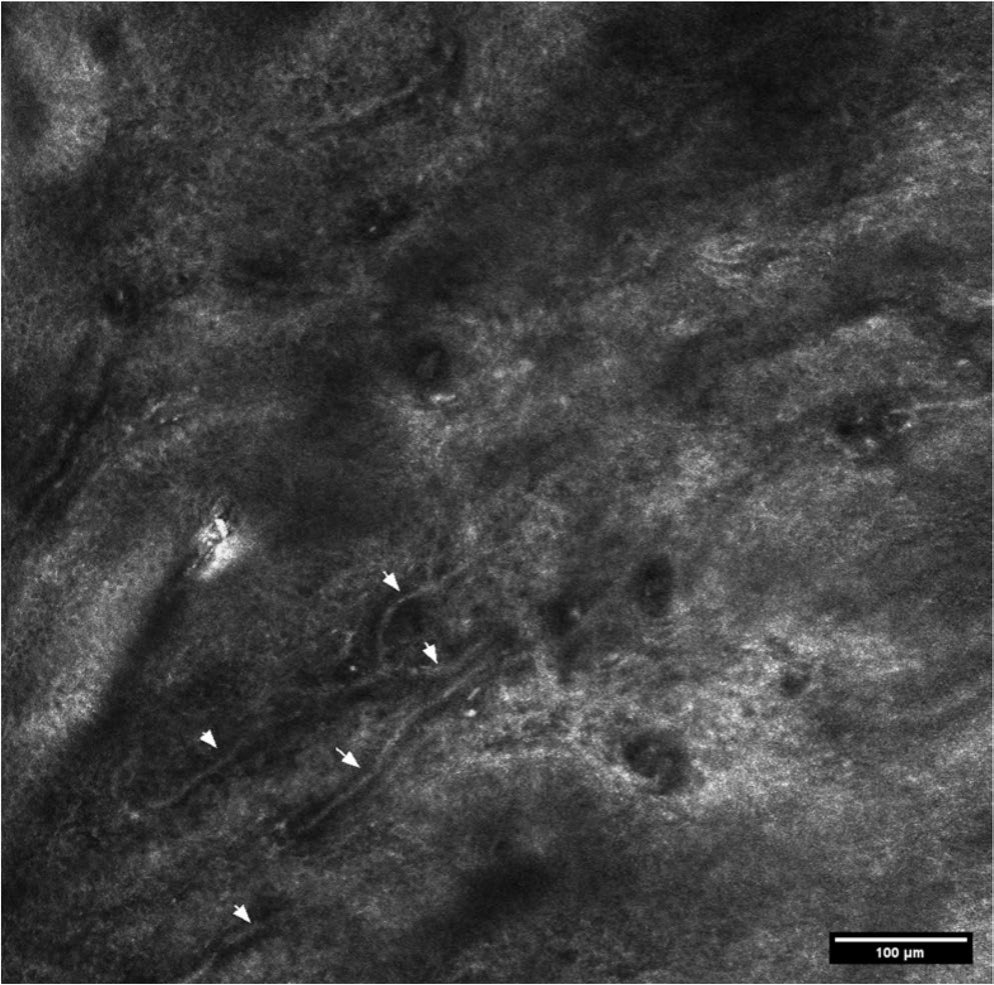
Identification of SVD in PWS patients via RCM. A representative RCM image captured at the level of the superficial dermis. The white arrow indicates one of the initial appearances of vasculature encountered when moving down from the epidermis, defining the SVD as 83.33 μm. Scale bar: 100 μm. Abbreviations: PWS, port wine stain; PDL, pulsed dye laser; RCM, reflectance confocal microscopy; SVD, superficial vessel depth

**Fig. 3:**
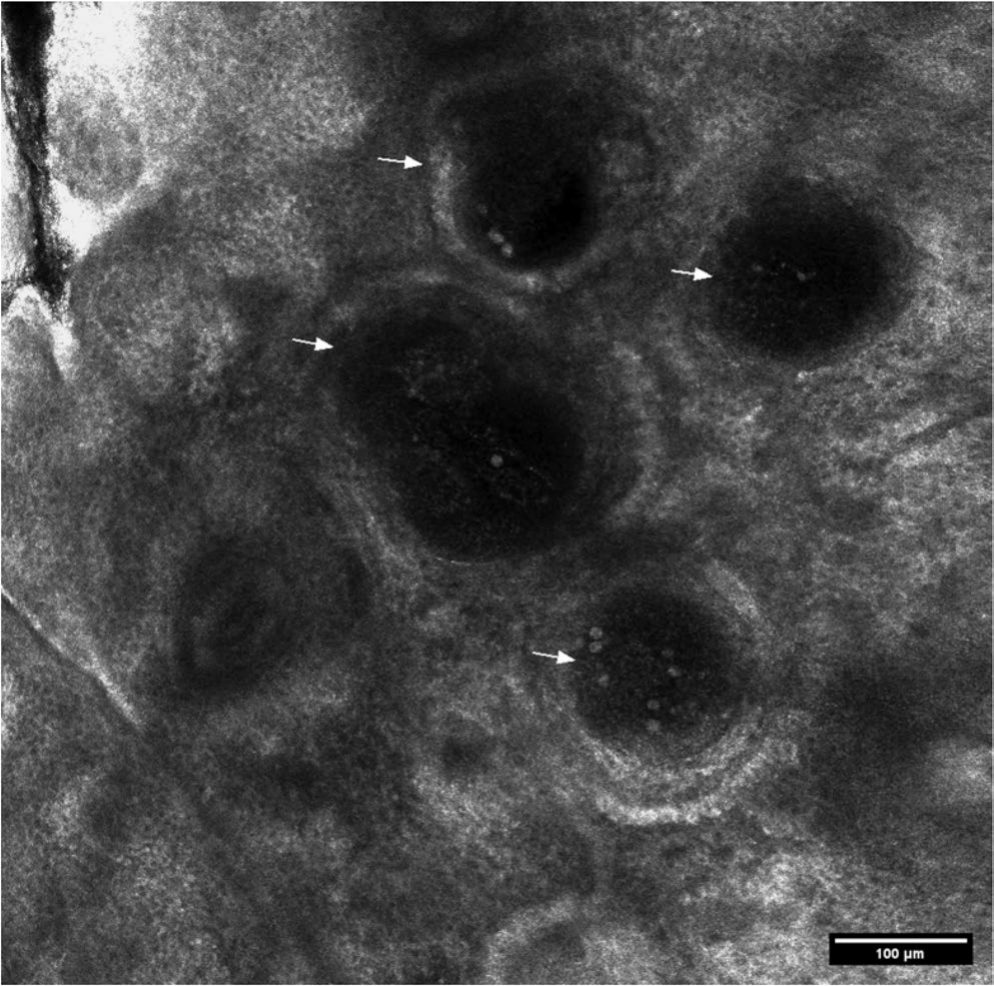
Calculation of VD in PWS patients via RCM. A representative RCM image (750 μm × 750 μm) of the superficial dermis displays vasculature characteristic of port wine stain, with four vessels indicated by white arrows. The depth was 87.23 μm. The VD was calculated as 4 vessels / (0.75 mm × 0.75 mm) = 7.11 vessels/mm². Scale bar: 100 μm. Abbreviations: PWS, port wine stain; RCM, reflectance confocal microscopy; MVD, maximum vessel diameter

**Fig. 4:**
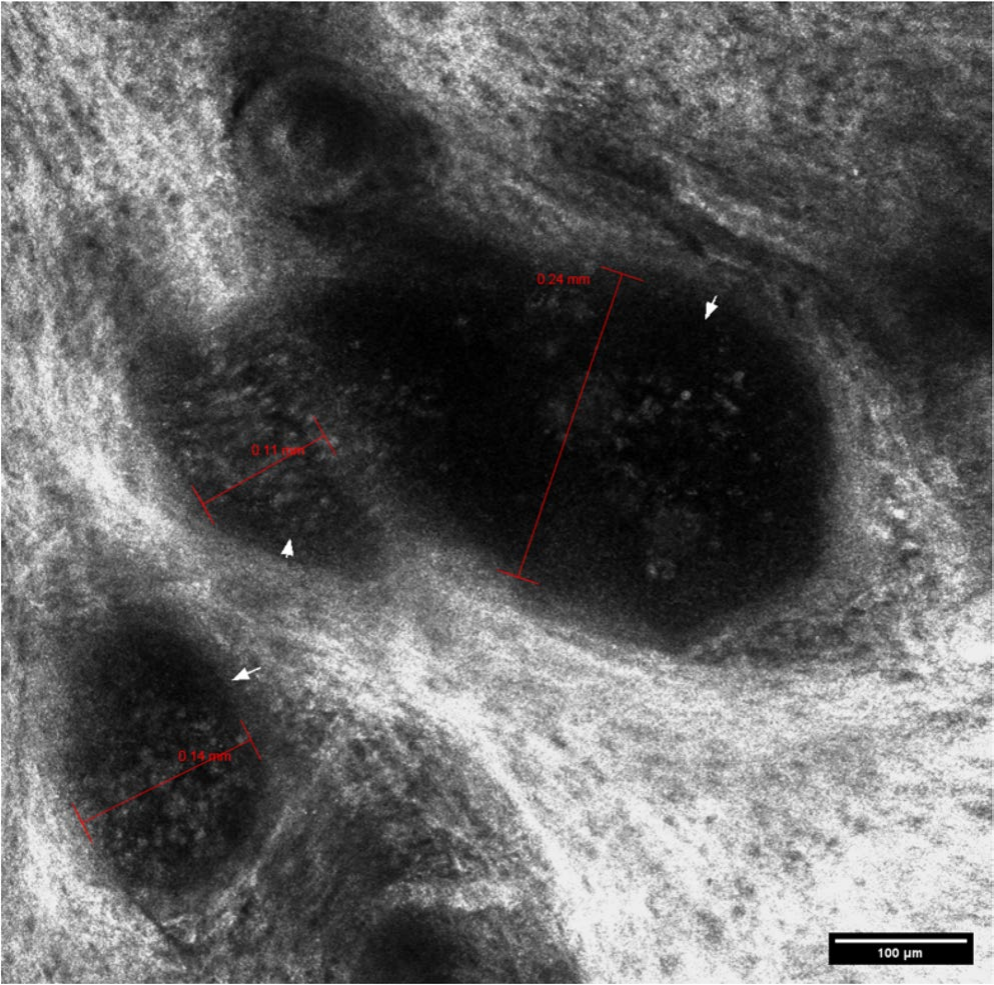
In vivo measurement of the MVD in PWS patients via RCM. A representative RCM image shows characteristic dilated vessels (indicated by white arrows) in the superficial dermis. The diameter of a target vessel was quantified using the built-in measurement tool (precision: 0.01 mm), as denoted by the red line. The MVD observed in this field was 240 μm, and the depth was 102.86 μm. Scale bar: 100 μm. Abbreviations: PWS, port wine stain; RCM, reflectance confocal microscopy; VD, vessel density

Using the live-video mode of the VivaScope 3000 system, scanning began at the skin surface (0 μm) and progressed downward in ~ 3.05-µm increments. The presence of flowing erythrocytes was confirmed by direct real-time visualization of intravascular cell movement during dynamic video scanning. Scanning continued until the most superficial blood vessel containing flowing erythrocytes was visualized; the depth at this level was defined as the superficial vessel depth (SVD) (Fig. 2). Scanning was then extended to deeper layers, and three images with the greatest density of dilated PWS vessels were captured for detailed imaging. For vessel density (VD) (Fig. 3), five images (0.75 mm × 0.75 mm each) obtained from layers with the highest concentration of dilated vessels were selected for counting. These same layers were used for vessel diameter measurements. Vessel diameter was determined using the system’s built-in measurement tool; all clearly visible vessels in the three representative deep-layer images were measured, and the maximum vessel diameter (MVD) for each lesion was defined as the largest diameter identified across all measured vessels (Fig. 4). The number of measurable vessels varied by lesion.

### Clinical outcome evaluation

Standardized digital photographs were taken for all patients before and after PDL treatment. Follow-up was scheduled at approximately 2 months (± 2-week variation). For treatment-naïve patients, the clearance rate after PDL treatment was visually evaluated by two blinded, independent, and experienced clinicians. In cases of disagreement, the final grading would be determined by a more senior clinician.

The primary outcome, the clearance rate after PDL treatment, was graded using a standard quartile grading scale: Grade 1 = 1–25% (no response), Grade 2 = 26–50% (improvement), Grade 3 = 51–75% (response), and Grade 4 ≥ 75% (complete clearance). Treatment was considered effective when the response was Grade ≥ 2, and ineffective when it was Grade 1. The Grade ≥ 2 threshold was selected based on prior literature in PDL outcome research, where Grade 1 (≤ 25% clearance) is widely accepted as representing a clinically meaningful lack of response [18]. 

### Statistical analysis

The data were analyzed using SPSS 22.0 (IBM Corp., Armonk, NY, USA) and GraphPad Prism 9.0 (GraphPad Software, San Diego, CA, USA). Continuous variables are presented as medians with interquartile ranges, and categorical variables are presented as frequencies and percentages. Differences in SVD, MVD, and VD among groups stratified by prior PDL treatment sessions were assessed using the Kruskal-Wallis test, followed by Dunn’s post hoc test with Bonferroni correction for multiple comparisons. For post hoc pairwise comparisons, Bonferroni-adjusted P values were reported. Comparisons between effective and ineffective groups were performed using the Mann-Whitney U test for continuous variables and Fisher’s exact test for categorical variables. In the longitudinal subset with paired pre- and post-treatment RCM data, within-group changes in RCM parameters were assessed using the Wilcoxon signed-rank test, and between-group differences in the magnitude of change were compared using the Mann-Whitney U test. For all analyses, *P* < 0.05 was considered statistically significant.

## Results

A total of 240 patients met the inclusion criteria and were enrolled in the cross-sectional RCM analysis. The cohort included 84 males and 156 females, with a mean age of 18.96 years (range, 0.15-59 years). All patients had Fitzpatrick skin phototypes III-V, and the mean number of prior PDL treatments was 9.82 sessions (range, 0–99).

Among the 240 patients, 54 were treatment-naïve before PDL treatment. Of these, 25 completed the 2-month follow-up after their initial PDL session and were included in the efficacy analysis. The remaining 29 patients were excluded because of loss to follow-up (*n* = 18), early return for re-treatment before the scheduled follow-up assessment (*n* = 8), or incomplete photographic records (*n* = 3). Among the 25 patients in the efficacy cohort, 14 had paired pre- and post-treatment RCM data and were included in the longitudinal analysis, comprising 7 effective and 7 ineffective responders. The remaining 11 patients had no follow-up RCM imaging available, including 6 effective and 5 ineffective responders. The patient flow diagram is presented in Fig. 1, and baseline characteristics are summarized in Table 1.

**Table 1:** Demographic characteristics of patients

Variables	Value
Total	*N* = 240
Age	0.15-59 years (mean 18.96)
Gender	Male 84 (35%) Female 156 (65%)
Lesion location	Facial (100%)
Modalities of treatment	PDL (spot 10 mm, pulse width: 0.45–1.5 ms; energy: 6.0–8.25 J/cm^2^)
Number of treatments	0–99 (mean 9.82, median 4)
Prior PDL treatment history	Naïve 54 (22.5%), Treated: 186 (77.5%)

Abbreviations: *PDL* pulsed dye laser

The overall median values of the RCM-derived vascular parameters were 76.01 μm for SVD (IQR, 64.47–89.40 μm), 40 μm for MVD (IQR, 30–60 μm), and 7.30/mm² for VD (IQR, 4.75–10.88/mm²). Patients were stratified into five groups according to prior PDL treatment history: 0, 1–4, 5–10, 11–30, and ≥ 31 sessions. Significant differences in SVD and MVD, but not VD, were observed among groups stratified by prior PDL treatment history, as summarized in Table 2.

**Table 2:** Comparison of pre-treatment vascular characteristics by prior PDL treatment sessions

Variable	Group 1 (0 PDL) (*n* = 54)	Group 2 (1–4 PDL) (*n* = 87)	Group 3 (5–10 PDL) (*n* = 45)	Group 4 (11–30 PDL) (*n* = 27)	Group 5 (31–100 PDL) (*n* = 27)	*P*-value
Superficial Vessel Depth (SVD) (µm)						
Mean ± SD	67.30 ± 14.99	77.31 ± 17.51	77.13 ± 14.42	76.74 ± 12.58	88.11 ± 15.61	
Median (IQR)	67.46 (55.84, 78.25)	76.43 (64.45, 91.86)	76.80 (64.61, 88.63)	74.41 (68.85, 87.22)	90.36 (80.23, 97.32)	< 0.001
Max Vessel Diameter (MVD) (µm)						
Mean ± SD	80.74 ± 70.28	50.12 ± 34.25	42.00 ± 23.32	36.67 ± 15.69	35.93 ± 19.27	
Median (IQR)	60 (37.5, 100)	40 (30, 70)	30 (30, 55)	30 (20, 50)	30 (20, 50)	< 0.001
Vessel Density (VD) (/mm²)						
Mean ± SD	10.38 ± 6.36	8.50 ± 5.02	8.31 ± 4.74	7.23 ± 3.14	6.73 ± 4.30	
Median (IQR)	9.45 (4.88, 15.00)	6.89 (4.80, 11.36)	7.56 (4.81, 11.29)	7.36 (4.79, 9.05)	6.29 (4.13, 7.68)	0.109

a. Abbreviations: *PDL* pulsed dye laser

b. All RCM measurements were obtained immediately before each PDL session (pre-treatment)

**Fig. 5:**
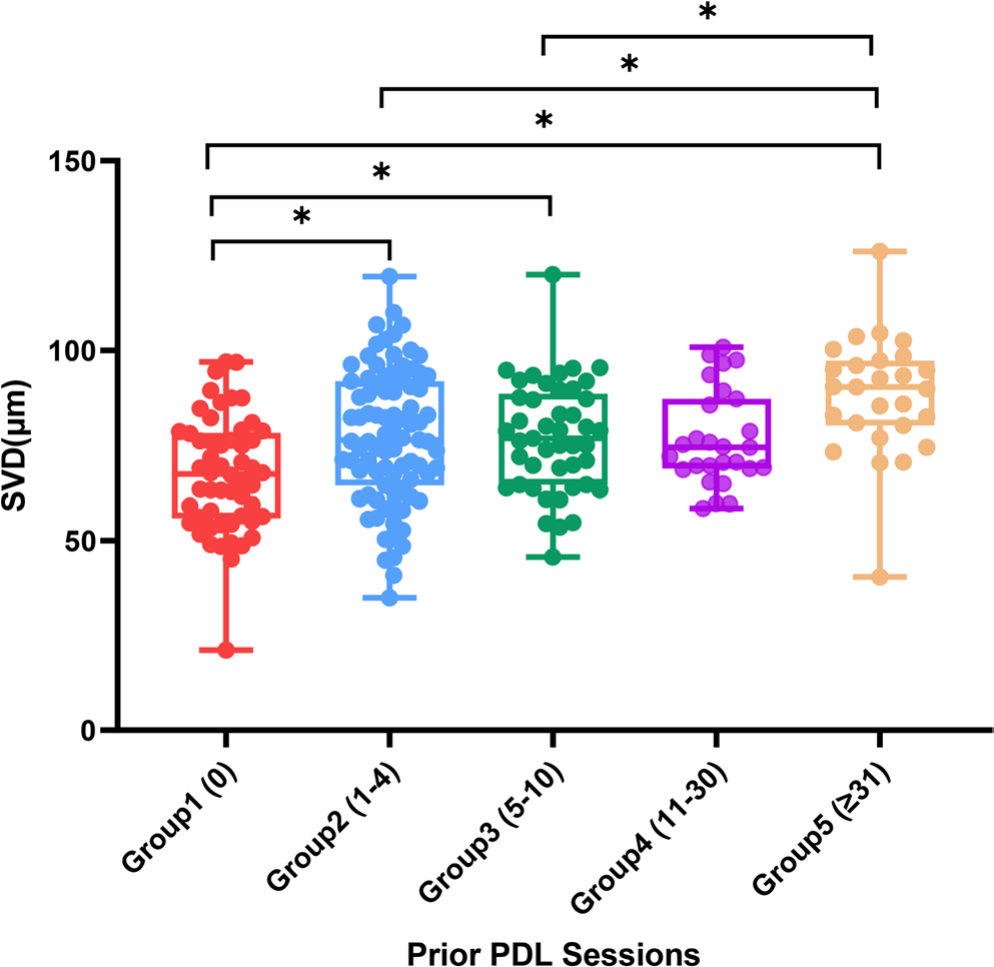
Box-and-whisker plots showing the distribution of SVD across different treatment session groups * *p* < 0.005 (Bonferroni-corrected). Abbreviations: SVD, superficial vessel depth; PDL, pulsed dye laser

SVD differed significantly across the five groups and increased with greater prior treatment burden (H(4) = 30.92, *P* < 0.001). Dunn–Bonferroni post hoc analysis showed that untreated lesions had significantly shallower SVD than lesions in the 1–4, 5–10, and ≥ 31 session groups. SVD in the ≥ 31 session group was also significantly greater than that in the 1–4 and 5–10 session groups. No significant differences were observed between the remaining group pairs (Fig. 5).

**Fig. 6:**
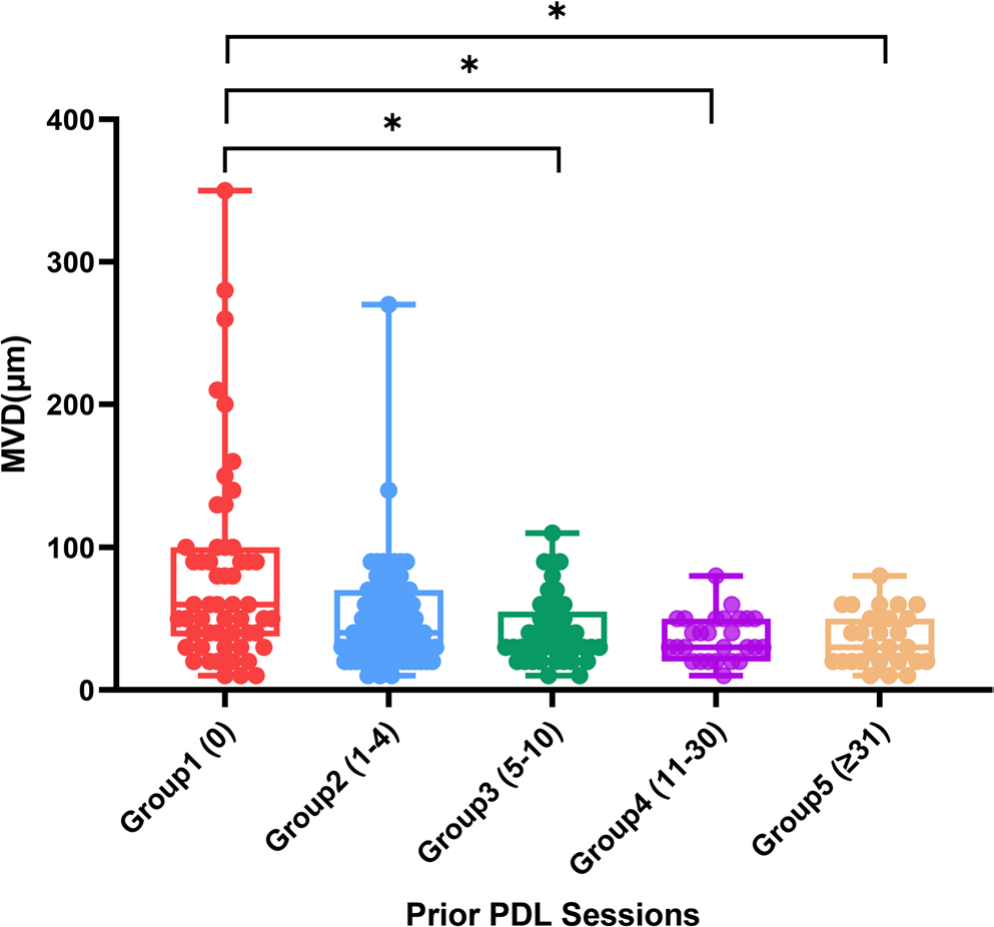
Distribution of MVD across port wine stain treatment groups * *p* < 0.005 (Bonferroni-corrected). Abbreviations: MVD, maximum vessel diameter ; PDL, pulsed dye laser

**Fig. 7:**
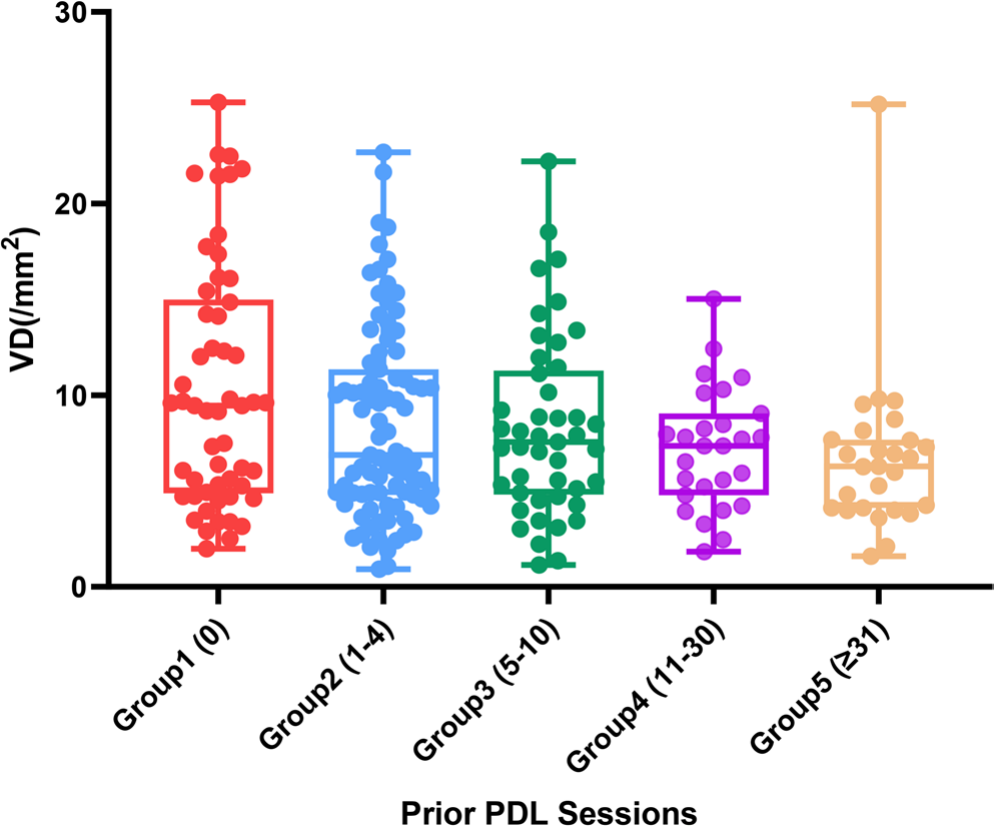
Distribution of VD across port wine stain treatment groups. Abbreviations: VD, vessel density; PDL, pulsed dye laser

MVD also differed significantly across groups (H(4) = 22.64, *P* < 0.001), showing a decreasing pattern with repeated PDL treatment (Fig. 6). Post hoc analysis showed that untreated lesions had significantly larger MVD than lesions in the 5–10, 11–30, and ≥ 31 session groups. No significant differences were observed between the other group pairs. In contrast, VD did not differ significantly among groups (H(4) = 7.56, *P* = 0.109) (Fig. 7).

Among the 25 treatment-naïve patients included in the efficacy analysis, 13 were classified as effective responders and 12 as ineffective responders. As summarized in Table 3, the two groups were comparable in baseline demographic and clinical characteristics, including age (*P* = 0.643), sex (*P* = 0.428), and lesion location (*P* = 0.845). Pretreatment MVD was significantly greater in the effective group than in the ineffective group (median [IQR], 90 [70–175] µm vs. 40 [30-47.5] µm; *P* < 0.001). No significant differences were observed in pretreatment SVD or VD between the two groups.

**Table 3:** Baseline demographic and clinical characteristics of the study stratified by treatment response

Characteristic	Overall (*N* = 25)	Ineffective (*n* = 12)	Effective (*n* = 13)	*P* Value
Demographics				
Age, years	19 (5–29)	15 (4–28)	23 (5–30)	*P* = 0.643
Sex, n(%)				*P* = 0.428
Male	10, 40%	6, 50%	4, 30.77%	
Female	15, 60%	6,50%	9, 69.23%	
Lesion, Location, n (%)				*P* = 0.845
V1	7, 28%	4, 33.33%	3, 23.08%	
V2	11, 44%	5, 41.67%	6, 46.15%	
V3	7, 28%	3, 25%	4, 30.77%	
RCM Parameters				
SVD, µm	65.84 (55.96–75.59)	67.63 (58.20-82.58)	65.84 (51.75–72.77)	*P* = 0.480
MVD, µm	60 (40–110)	40 (30-47.5)	90 (70–175)	*P* < 0.001
VD, /mm^2^	9.59 (5.51–14.49)	7.79 (4.98–15.19)	9.63 (5.67–14.49)	*P* = 0.624

a. *p* values for continuous variables were calculated using the Mann–Whitney U test

b. *p* values for categorical variables were calculated using Fisher’s exact test

c. Data are presented as median (interquartile range) or n (%), as appropriate

d. Abbreviations: *PWS* port wine stain, *PDL* pulsed dye laser, *RCM* reflectance confocal microscopy, *V1* ophthalmic branch of the trigeminal nerve, *V2* maxillary branch of the trigeminal nerve, *V3* mandibular branch of the trigeminal nerve

To further examine the association between MVD and treatment efficacy, patients were stratified according to baseline MVD using a cutoff value of 40 μm. Patients with MVD > 40 μm showed a significantly higher response rate than those with MVD ≤ 40 μm. All effective responders had an MVD > 40 μm, whereas 9 of 12 ineffective responders had an MVD ≤ 40 μm. The distribution differed significantly between groups (*P* < 0.001, Fisher’s exact test), as shown in Table 4.

**Table 4:** Association between maximum vessel diameter (MVD) and clinical efficacy of PDL treatment

MVD Group, µm	*n*, %	Ineffective	Effective	*P* Value
≤ 40	9, 36%	9, 75%	0, 0%	*P* < 0.001
> 40	16, 64%	3, 25%	13, 100%	

a. *p* value was calculated using Fisher’s exact test

b. Abbreviations: *PDL* pulsed dye laser

**Fig. 8:**
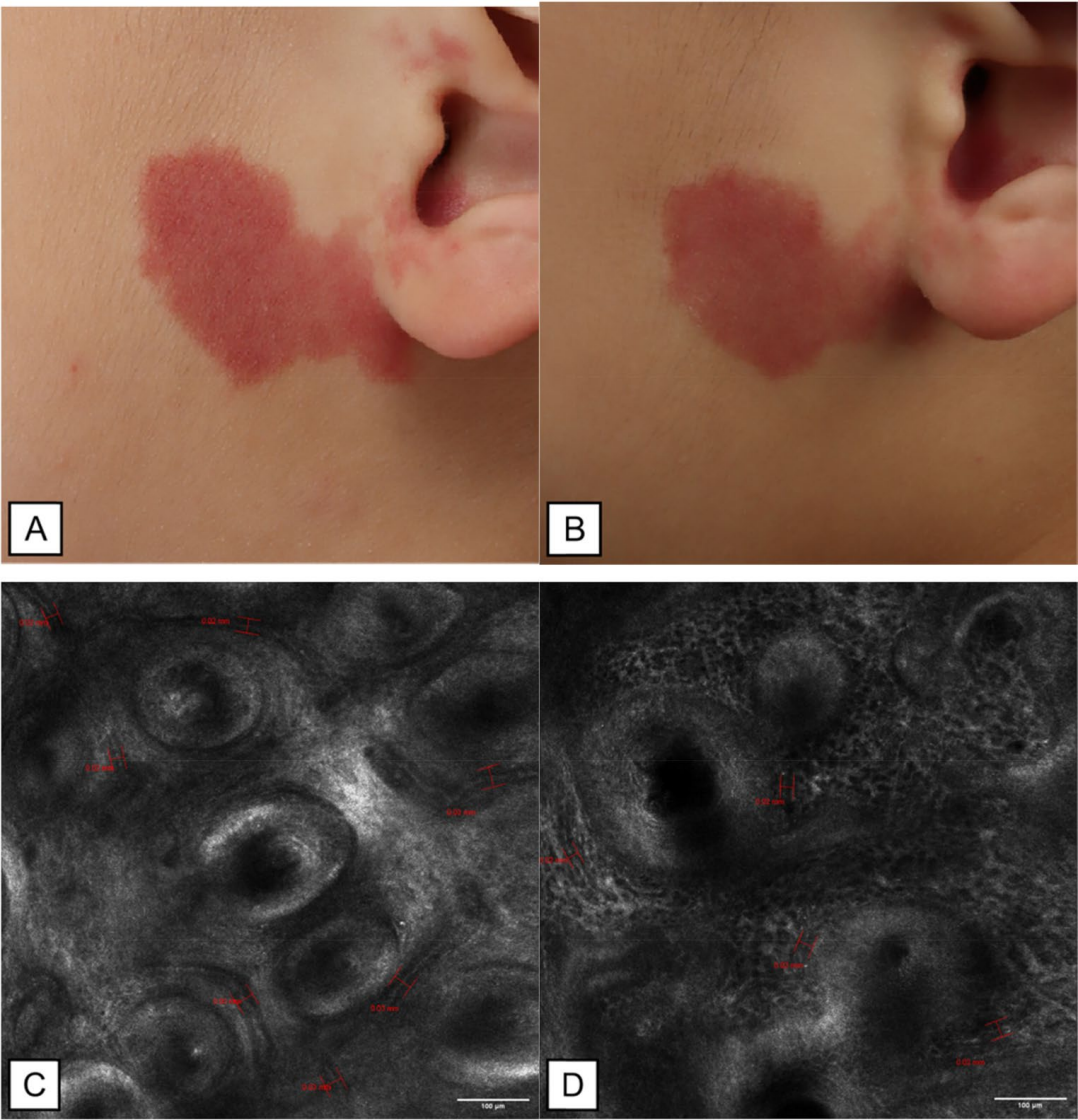
Representative clinical and RCM images of another PWS patient with a suboptimal response to laser treatment. (**A**) Baseline clinical photograph before the first treatment. (**B**) Clinical photograph at the 2-month follow-up after a single PDL session, showing less than 25% clearance. (**C**) RCM image taken before treatment, demonstrating an MVD of 30 μm; the depth was 87.89 μm. Scale bar: 100 μm. (**D**) RCM image taken 2 months after PDL treatment, demonstrating an MVD of 20 μm; the depth was 76.35 μm. Scale bar: 100 μm. Abbreviations: RCM, reflectance confocal microscopy; PWS, port wine stain; PDL, pulsed dye laser; MVD, maximum vessel diameter

**Fig. 9:**
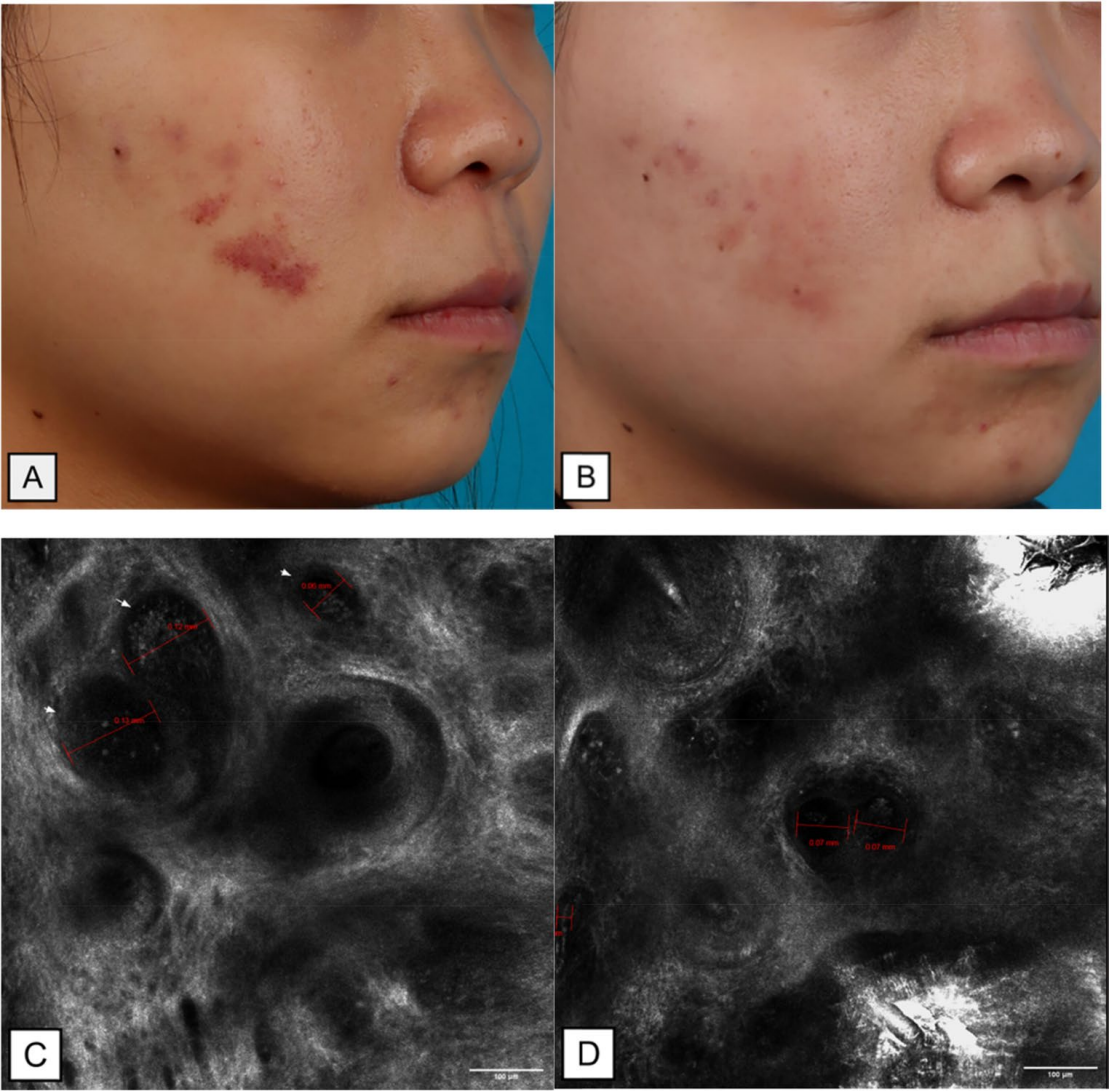
Representative clinical and RCM images of a port wine stain patient with a favorable response to laser treatment. (**A**) Baseline clinical photograph before the first treatment. (**B**) Clinical photograph at the 2-month follow-up after a single PDL session, showing clearance exceeding 75%. (**C**) RCM image taken before treatment, demonstrating an MVD of 130 μm; the depth was 91.79 μm. Scale bar: 100 μm. (**D**) RCM image taken 2 months after PDL treatment, demonstrating an MVD of 80 μm; the depth was 85.93 μm. Scale bar: 100 μm. Abbreviations: RCM, reflectance confocal microscopy; PWS, port wine stain; PDL, pulsed dye laser; MVD, maximum vessel diameter

Representative cases are shown in Figs. 8 and 9. One patient with a baseline MVD of 30 μm showed less than 25% clearance after a single PDL session. In contrast, another patient with a baseline MVD of 130 μm achieved more than 75% clearance after treatment. These cases illustrate the association between larger pretreatment vessel diameter and more favorable clinical improvement.

In the longitudinal subset, MVD decreased significantly after a single PDL session in the effective group, from 90 μm to 50 μm (*P* < 0.05), whereas no significant changes were observed in SVD or VD. In the ineffective group, no significant changes were observed in SVD, MVD, or VD after treatment. The magnitude of MVD reduction was significantly greater in the effective group than in the ineffective group (-30 μm vs. -10 μm, *P* < 0.05), whereas changes in SVD and VD did not differ significantly between groups. These results are summarized in Tables 5, 6 and 7.

**Table 5:** Pre- and post-treatment RCM parameters in the effective group (*n* = 7)

	Pre-SVD(µm)	Post-SVD(µm)	Pre-MVD(µm)	Post-MVD(µm)	Pre-VD(/mm²)	Post-VD(/mm²)
Median	69.52	72.89	90	50	8.89	5.33
p-value*		0.813		0.031		0.609

Abbreviations: *RCM* reflectance confocal microscopy, *SVD* superficial vessel depth, *MVD* maximum vessel diameter, *VD* vessel density. *p values indicate paired comparisons between pre- and post-treatment parameters

**Table 6:** Pre- and post-treatment RCM parameters in the ineffective group (*n* = 7)

Patient	Pre-SVD(µm)	Post-SVD(µm)	Pre-MVD(µm)	Post-MVD(µm)	Pre-VD(/mm²)	Post-VD(/mm²)
Median	74.86	75.41	40	30	12.44	7.11
p-value*		1.000		1.000		0.094

Abbreviations: *RCM* reflectance confocal microscopy, *SVD* superficial vessel depth, *MVD* maximum vessel diameter, *VD* vessel density. *p values indicate paired comparisons between pre- and post-treatment parameters

**Table 7:** Between-group comparison of RCM parameter changes following PDL treatment

Parameter	Effective Group (*n* = 7) Median Change	Ineffective Group (*n* = 7) Median Change	*p*-value
SVD (µm)	−2.25	+ 0.55	1.000
MVD (µm)	−30	−10	0.020
VD (/mm²)	−1.78	−3.56	0.535

*p values were calculated using the Mann-Whitney U test. Median change = median of individual post-pre differences

## Discussion

The primary finding of this exploratory, retrospective study is that pretreatment MVD is associated with therapeutic response to PDL in patients with PWS. Lesions with an MVD ≤ 40 μm generally showed poor responses to the first treatment, whereas those with an MVD > 40 μm were more likely to achieve effective clearance. This observation is consistent with previously reported theoretical predictions [19] suggesting that vessel diameter may serve as a potential indicator of PDL responsiveness. Although the 40 μm cutoff was informed by prior literature—which has reported that PWS with pretreatment capillary diameters > 40 μm are more likely to respond to PDL than those with smaller diameters [10]—it was not pre-specified in the study protocol and was not validated in an independent cohort. Notably, the median MVD of the ineffective group in our cohort independently converged at this same value, lending further support to its relevance. Nevertheless, the risk of overfitting cannot be excluded, and the observed complete separation in response rates should be interpreted with caution. This finding was further corroborated by a supplementary pre- and post-treatment RCM analysis in a subset of 14 patients with available repeat imaging. In the effective group, MVD decreased significantly following a single PDL session, whereas no significant change was observed in the ineffective group. Furthermore, the magnitude of MVD reduction was significantly greater in the effective group than in the ineffective group, suggesting that larger vessels not only predict better treatment response at baseline but also undergo greater structural remodeling following PDL treatment. No significant differences were observed in SVD or VD between the two groups following treatment, consistent with the primary cross-sectional findings. These findings should therefore be regarded as hypothesis-generating and require confirmation in a larger, prospective, independent cohort.

This preliminary conclusion is also supported by our cross-sectional data. Among patients who had undergone more than five PDL sessions, the median MVD stabilized at approximately 30 µm—within the ‘poorly responsive’ range identified in our study (≤ 40 μm)—suggesting that patients with greater prior PDL exposure showed smaller MVD, which may reflect preferential reduction of larger, more responsive vessels over repeated treatments, although this cross-sectional pattern cannot establish causality. This pattern may help explain why a therapeutic plateau often appears after several PDL sessions.The poor treatment response of small vessels may be explained by their photothermal characteristics [20]. Their higher surface-to-volume ratio results in both a shorter thermal relaxation time and faster heat dissipation, making it more difficult to achieve the sustained temperature elevation required for irreversible photocoagulation with standard PDL pulse durations [20, 21].

It is worth noting that some studies have reported findings inconsistent with ours. For example, Fu et al. reported that laser-resistant PWS blood vessels had larger diameters and were located deeper in the skin [16]. This apparent discrepancy likely reflects fundamental differences in study design and measurement methodology rather than a true biological contradiction. First, Fu et al. evaluated vascular characteristics in patients who had undergone 4–6 sessions of multiplex 585/1,064 nm laser treatment, whereas our study assessed first-session PDL response in treatment-naïve patients—two fundamentally different clinical questions at different stages of disease management. Second, Fu et al. reported average vessel diameter measured across all visible vessels, whereas our study used maximum vessel diameter (MVD) as the primary parameter; these two metrics capture different aspects of vascular architecture and are not directly comparable. Additionally, histological studies have reported conflicting patterns, with some finding deeper vessels associated with resistance and others emphasizing vessel diameter [11, 22].

A critical limitation of this study is the effective imaging depth of RCM (~ 150 μm in our cohort), which is shallower than the ~ 250 μm reported in some prior studies [23] and may not capture deeper dermal vessels known to contribute to PDL resistance. Deeper imaging modalities—including optical coherence tomography (OCT), which can image to ~ 1–2 mm, and high-frequency ultrasound—have the potential to visualize a more complete spectrum of the dermal vascular network. OCT in particular has emerged as a valuable noninvasive tool for PWS vascular mapping, providing detailed characterization of vessel depth, diameter, and density, and enabling individualized pre- and post-treatment parameter adjustments [24]. Vessels residing below the RCM imaging threshold may have larger diameters or different structural characteristics that could influence PDL response, and it is therefore plausible that the absence of predictive value for SVD and VD reflects technical rather than biological limitations. This interpretation should therefore be understood as constrained to the superficial vascular compartment accessible by RCM, rather than the entire dermal vasculature. Future studies integrating RCM with deeper imaging modalities such as OCT or high-frequency ultrasound would provide a more comprehensive assessment of the vascular predictors of PDL efficacy in PWS [25].

Based on these observations, we speculate that treatment-resistant PWS may be associated with a shift toward smaller, deeper, or faster-cooling vessels after repeated laser exposure. As treatment sessions increase, larger vessels tend to be removed first, while smaller, deeper, or faster-cooling vessels are more likely to remain and gradually become predominant, forming a morphologically resistant phenotype consistent with the clinical plateau commonly seen after around five PDL sessions [10, 26]. Currently, there is no unified definition of ‘resistant PWS,’ with variable criteria used in the literature (e.g., lack of further improvement after 8–15 PDL sessions) [27, 28]. Our findings may help generate candidate imaging features for future definitions of treatment-resistant PWS, such as poor response after multiple PDL sessions combined with small-vessel predominance on RCM; however, these features require validation in larger, prospective studies.

## Limitations

Several limitations should be acknowledged. This was a small retrospective exploratory study with an efficacy cohort of only 25 patients, limiting statistical power. The 40 μm MVD cutoff was derived post hoc and was not validated in an independent cohort. The cross-sectional comparison across prior-treatment groups cannot establish within-patient vascular evolution over repeated PDL sessions. The limited imaging depth of RCM may have restricted assessment of deeper dermal vessels, and individualized treatment parameters may have introduced confounding. Finally, treatment response was assessed after a single PDL session at a 2-month follow-up using physician-graded clearance; long-term multi-session outcomes and patient-reported measures were not evaluated. Larger prospective studies with standardized protocols and independent validation are required.

## Conclusion

In this retrospective exploratory study, baseline MVD assessed by RCM was associated with PDL response in patients with PWS. Lesions with larger baseline vessels, particularly those with MVD > 40 μm, tended to show more favorable clinical improvement, whereas smaller vessels were associated with poorer response. Cross-sectional findings further suggested that repeated PDL treatment may preferentially reduce larger vessels, leaving smaller vessels predominant. Given the small efficacy cohort and post hoc cutoff, these findings should be considered hypothesis-generating and require validation in larger prospective cohorts before clinical implementation. Future studies combining RCM with deeper imaging modalities may help refine vessel diameter-guided treatment strategies for PWS.

## Data Availability

Data are available from the corresponding author upon reasonable request.
